# Supplementary figures and images for: Design of a Multi-Epitope Vaccine against Histoplasma capsulatum through Immunoinformatics Approaches
Source: J Fungi (Basel). 2024 Jan 5;10(1):43. doi: 10.3390/jof10010043 (PMC10817582; doi:10.3390/jof10010043)

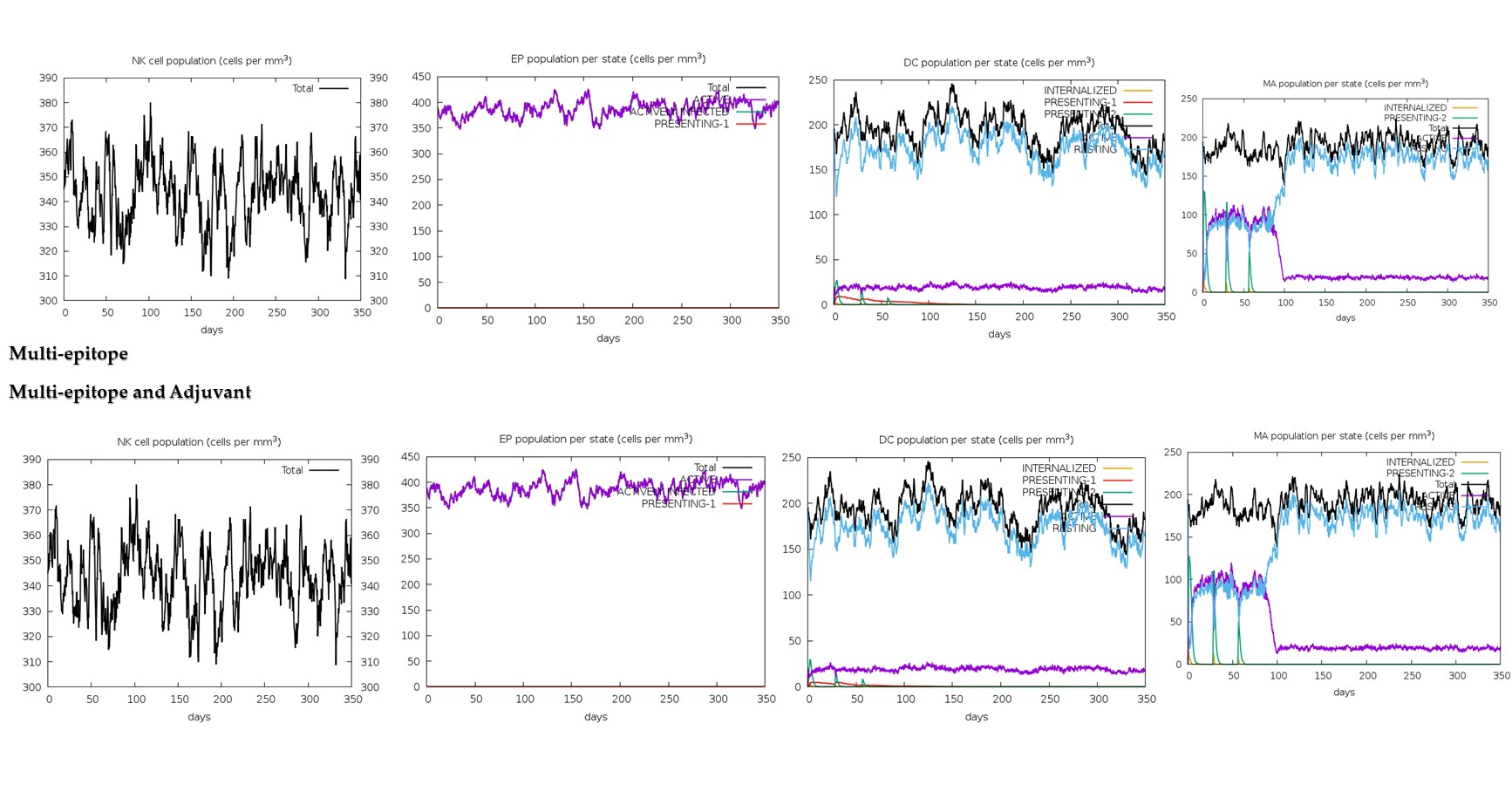

Supplement: Supplementary file 1 [file jof-10-00043-s001.zip › Supplementary_figure_S1.jpg]
